# Neuroimmune crosstalk in chronic neuroinflammation: microglial interactions and immune modulation

**DOI:** 10.3389/fncel.2025.1575022

**Published:** 2025-04-07

**Authors:** Ludmila Müller, Svetlana Di Benedetto

**Affiliations:** Max Planck Institute for Human Development, Center for Lifespan Psychology, Berlin, Germany

**Keywords:** neuroimmune crosstalk, neuroinflammation, microglia, astrocytes, neurons, immune cells, cytokines, neurodegenerative diseases

## Abstract

Neuroinflammation is a fundamental feature of many chronic neurodegenerative diseases, where it contributes to disease onset, progression, and severity. This persistent inflammatory state arises from the activation of innate and adaptive immune responses within the central nervous system (CNS), orchestrated by a complex interplay of resident immune cells, infiltrating peripheral immune cells, and an array of molecular mediators such as cytokines, chemokines, and extracellular vesicles. Among CNS-resident cells, microglia play a central role, exhibiting a dynamic spectrum of phenotypes ranging from neuroprotective to neurotoxic. In chronic neurodegenerative diseases, sustained microglial activation often leads to the amplification of inflammatory cascades, reinforcing a pathogenic cycle of immune-mediated damage. Intercellular communication within the inflamed CNS is central to the persistence and progression of neuroinflammation. Microglia engage in extensive crosstalk with astrocytes, neurons, oligodendrocytes, and infiltrating immune cells, shaping both local and systemic inflammatory responses. These interactions influence key processes such as synaptic pruning, phagocytosis, blood–brain barrier integrity, and cytokine-mediated signaling. Understanding the mechanisms of cell–cell signaling in this context is critical for identifying therapeutic strategies to modulate the immune response and restore homeostasis. This review explores the key players in CNS neuroinflammation, with a focus on the role of microglia, the molecular pathways underlying intercellular communication, and potential therapeutic approaches to mitigate neuroinflammatory damage in chronic neurodegenerative diseases.

## 1 Introduction

Neuroinflammation is a hallmark of many chronic neurodegenerative diseases, including Alzheimer’s Disease (AD), Parkinson’s Disease (PD), and Multiple Sclerosis (MS). It is characterized by the activation of immune responses within the CNS, often persisting in a dysregulated manner and contributing to progressive neuronal dysfunction and degeneration (Kempuraj et al., 2016; Matejuk et al., 2021; Mayne et al., 2020; Müller and Di Benedetto, 2024). Unlike acute inflammation, which serves as a protective response to injury or infection, chronic neuroinflammation can lead to sustained activation of immune responses in the CNS, resulting in detrimental effects on neuronal health. The inflammatory milieu in these conditions is characterized by a complex interplay between CNS-resident cells, such as microglia and astrocytes, infiltrating peripheral immune cells, and a diverse array of molecular mediators, including cytokines, chemokines, and extracellular vesicles (Kwon and Koh, 2020; Matejuk et al., 2021; Müller et al., 2025; Nosi et al., 2021). These mediators not only regulate the inflammatory response but also affect the survival and functionality of neurons and other glial cells, creating a feedback loop that perpetuates inflammation and tissue damage. The sophisticated network of interactions underscores the CNS as a highly interconnected system, where cellular responses are dynamically modulated by local and systemic cues (Müller et al., 2025; Nosi et al., 2021).

Neuroinflammation plays a dual role in the CNS, acting as both a protective and pathological mechanism depending on the context. Under physiological conditions, microglia and astrocytes orchestrate a balanced immune response that facilitates tissue repair, synaptic remodeling, and clearance of toxic protein aggregates. Anti-inflammatory cytokines such as interleukin (IL)-10 and transforming growth factor (TGF-*β*) contribute to resolving acute inflammation and restoring homeostasis (Kolliker-Frers et al., 2021; Nosi et al., 2021). However, in chronic neurodegenerative diseases, persistent stimuli—such as amyloid-β accumulation in Alzheimer’s disease or *α*-synuclein aggregates in Parkinson’s disease—prolong microglial activation, leading to sustained production of pro-inflammatory mediators like tumor necrosis factor-alpha (TNF-α) and interleukin-1 beta (IL-1β). This ongoing inflammation can disrupt synaptic integrity, impair neurogenesis, and compromise neuronal survival. Moreover, an age-related decline in anti-inflammatory signaling may further exacerbate this imbalance, preventing the resolution of inflammation and contributing to disease progression (Gao et al., 2023; Shabbir et al., 2021; Thakur et al., 2023; Wu and Eisel, 2023; Yang et al., 2020; Yildirim-Balatan et al., 2024).

Microglia, the resident immune cells of the CNS, play a pivotal role in maintaining homeostasis and responding to injury or infection. Depending on the context, they can adopt phenotypes that range from neuroprotective to neurotoxic, responding to and influencing the surrounding cellular environment (Colonna and Butovsky, 2017; Leng and Edison, 2021; Li and Barres, 2018; Sierra et al., 2013). In chronic neurodegenerative diseases microglia can adopt maladaptive activation states, exacerbating inflammation and contributing to neuronal damage. Their interactions with other CNS cells, such as astrocytes and neurons, create signaling cascades that amplify neuroinflammation, often resulting in a shift from protective to neurotoxic outcomes. For instance, microglia–neuron interactions are central to synaptic remodeling and neuronal health, while microglia-astrocyte signaling can amplify inflammatory cascades (Lana et al., 2016; Lian et al., 2016; Lim et al., 2013; Matejuk et al., 2021). Moreover, the breakdown of the blood–brain barrier in neurodegenerative diseases facilitates the infiltration of peripheral immune cells, such as monocytes and T cells, further complicating the inflammatory landscape and creating additional therapeutic challenges (Takata et al., 2021).

Understanding the mechanisms of intercellular communication in chronic neuroinflammation is critical for unraveling the complex network of cellular interactions and molecular signaling pathways that drive disease progression (Müller et al., 2025; Nosi et al., 2021). Recent advancements in this field have shed light on the dual roles of microglia and astrocytes, the impact of dysregulated cytokine and chemokine signaling, and the influence of systemic inflammation on CNS health. By dissecting these pathways, researchers aim to identify novel therapeutic targets to modulate neuroinflammation, restore cellular homeostasis, and mitigate the long-term effects of these devastating diseases (Di Benedetto et al., 2017; DiSabato et al., 2016; Kwon and Koh, 2020; Nosi et al., 2021; Xue et al., 2024). This mini-review aims to provide a compact overview of intercellular communication in chronic neuroinflammatory diseases, emphasizing the role of microglia as central mediators of these processes. We also explore the molecular mediators that drive neuroinflammation, discuss their contributions to disease pathogenesis, and highlight emerging therapeutic strategies to modulate immune responses and restore CNS homeostasis. Understanding these complex interactions is critical for advancing our ability to mitigate the harmful effects of neuroinflammation in neurodegenerative diseases.

## 2 Cellular participants in neuroinflammation

**Figure 1:**
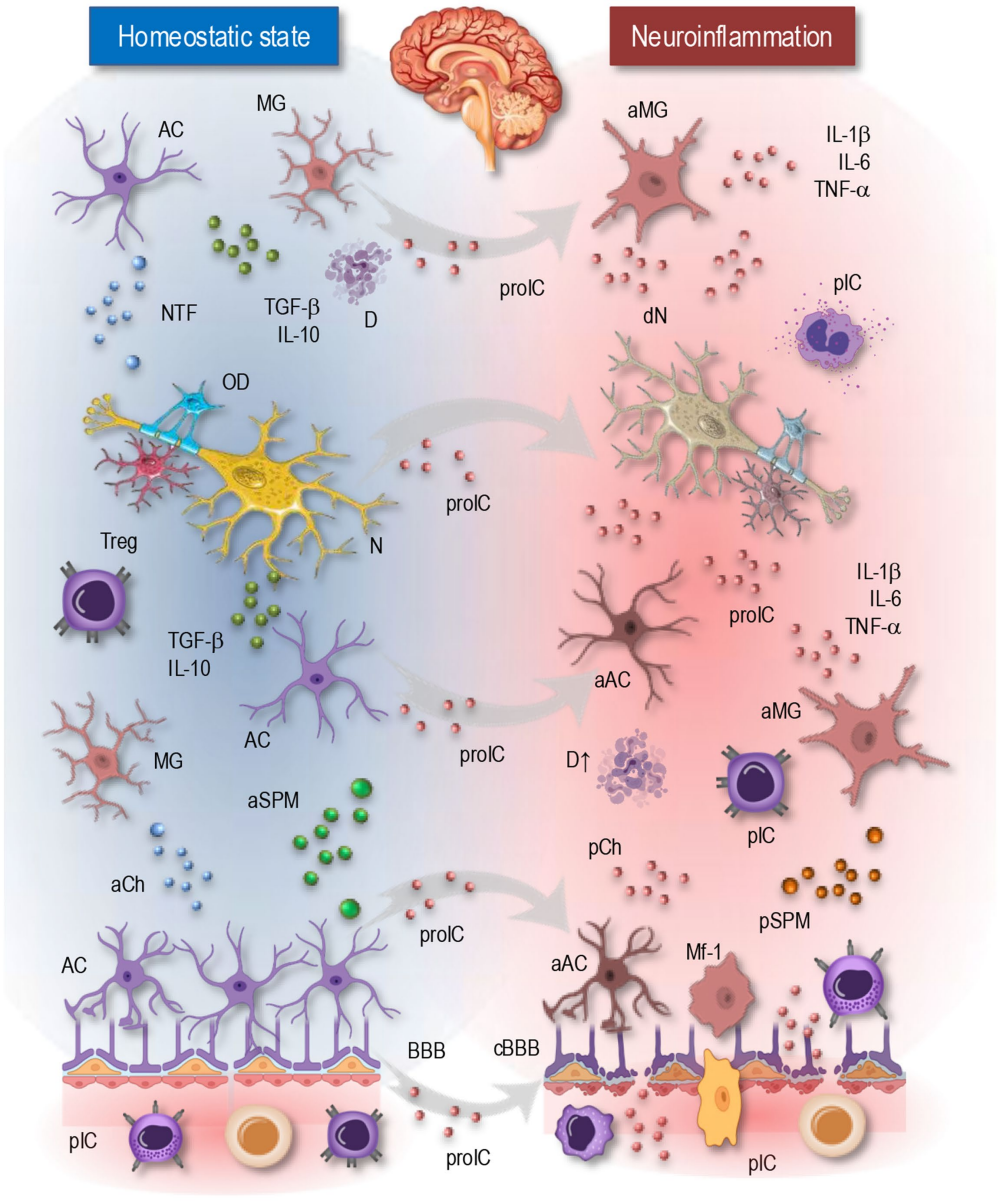
Cellular and molecular mediators of neuroinflammation. The left panel (blue background) illustrates cells and signaling molecules in the homeostatic state, where the blood–brain barrier remains intact, regulating immune interactions and maintaining CNS balance. The right panel (red background) depicts neuroinflammation, characterized by activated microglia, reactive astrocytes, BBB disruption, infiltrating immune cells, and increased pro-inflammatory mediators. MG, microglia; AC, astrocyte; NTF, neurotrophic factors; D, debris; TGF, transforming growth factor; IL, interleukin; OD, oligodendrocyte; N, neuron; Treg, regulatory T cell; aSPM, anti-inflammatory specialized pro-resolving mediators; aCh, anti-inflammatory chemokines; aMG, activated microglia; aAC, activated astrocyte; TNF, tumor necrosis factor; pIC, peripheral immune cells; dN, degenerating neuron; proIC, pro-inflammatory cytokines; pCh, pro-inflammatory chemokines; pSPM, pro-inflammatory SPM; Mf-1, macrophage type 1; BBB, blood–brain-barrier; cBBB, compromised blood–brain-barrier.

Neuroinflammation involves a diverse array of cellular players, both resident within the CNS and infiltrating from the periphery (Figure 1). These cells engage in dynamic interactions, shaping the inflammatory environment and influencing disease progression (Kwon and Koh, 2020; Labzin et al., 2018; Lee et al., 2023; Matejuk et al., 2021). While some immune responses serve protective roles, prolonged or dysregulated activation can lead to neuronal dysfunction and degeneration. In this section, we provide a brief overview of the key cellular players involved in these processes.

Microglia are the primary immune cells of the CNS, serving as the first line of defense against pathogens and injury. In their homeostatic state (Figure 1, left), microglia continuously surveil the microenvironment, clearing debris and maintaining neuronal integrity. However, in chronic neuroinflammatory conditions (Figure 1, right), they can move into reactive states characterized by altered morphology, secretion of pro-inflammatory cytokines such as TNF-*α* and IL-1*β*, and excessive synaptic pruning. This persistent activation contributes to neurotoxicity and exacerbates disease pathology in conditions like AD and PD. Despite their central role in neuroinflammation, microglia also possess neuroprotective functions, releasing anti-inflammatory factors such as IL-10 and TGF-β, which help resolve inflammation and promote tissue repair. The balance between these opposing roles is critical in determining the outcome of neuroinflammatory responses (Colonna and Butovsky, 2017; Di Benedetto et al., 2017; Kwon and Koh, 2020; Labzin et al., 2018).

Astrocytes serve as key regulators of CNS homeostasis, supporting neuronal function, maintaining the blood–brain barrier (BBB), and modulating synaptic activity. In response to inflammatory stimuli, astrocytes undergo reactive changes, leading to the release of pro-inflammatory cytokines and chemokines that further activate microglia and recruit peripheral immune cells (Patani et al., 2023; Sanmarco et al., 2021; Santello et al., 2019; Song and Dityatev, 2018). While reactive astrocytes can exert neuroprotective effects by producing neurotrophic factors and scavenging toxic molecules, persistent activation can drive chronic inflammation and contribute to neurodegeneration. Crosstalk between microglia and astrocytes plays a pivotal role in shaping the inflammatory landscape, with microglial-derived signals influencing astrocytic responses and vice versa (Kwon and Koh, 2020; Müller et al., 2025).

Oligodendrocytes and oligodendrocyte precursor cells (OPCs) are primarily responsible for myelination and axonal support. In neuroinflammatory diseases such as MS, oligodendrocytes are highly susceptible to immune-mediated damage, leading to demyelination and impaired neuronal function. OPCs have the potential to differentiate into new oligodendrocytes and contribute to remyelination; however, inflammatory conditions often hinder their regenerative capacity. The interactions between oligodendrocytes, microglia, and astrocytes are crucial in determining the extent of myelin repair versus degeneration (Lopez-Muguruza and Matute, 2023; Matejuk et al., 2021; Yeung et al., 2019).

Neurons are not passive bystanders in neuroinflammation but actively participate in immune signaling. Under inflammatory conditions, neurons express cytokine and chemokine receptors, allowing them to respond to immune signals and influence glial cell activity. However, sustained exposure to inflammatory mediators can disrupt synaptic function, impair neuronal plasticity, and ultimately lead to neurodegeneration. Microglial and astrocytic responses to neuronal distress signals further amplify inflammation, creating a feedback loop that sustains disease progression (Badimon et al., 2020; La Rosa et al., 2020; Lana et al., 2016; Lim et al., 2013; Müller et al., 2025; Song and Dityatev, 2018).

During chronic neuroinflammation, peripheral immune cells gain access to the CNS, particularly when the BBB becomes compromised. These infiltrating cells contribute to the inflammatory milieu and interact with resident glial cells, shaping the immune response. Monocytes and macrophages enter the CNS in response to chemokine gradients, differentiating into macrophage-like cells that can either support tissue repair or exacerbate neuroinflammation (Li and Barres, 2018; Mills et al., 2000; Wohleb et al., 2013). Unlike resident microglia, infiltrating macrophages exhibit distinct gene expression profiles and functional properties, making their contributions to neuroinflammation a subject of ongoing investigation (Bennett et al., 2018; Li and Barres, 2018).

T Cells, particularly CD4^+^ and CD8^+^ subsets, play a major role in modulating neuroinflammatory responses. In MS, autoreactive T cells infiltrate the CNS and drive demyelination through direct cytotoxic effects and cytokine production. In other neurodegenerative diseases, T-cell involvement is less well understood but has been implicated in modulating microglial activation and shaping inflammatory responses. Regulatory T cells (Tregs) have acquired interest as potential therapeutic targets due to their ability to suppress excessive inflammation and promote tissue repair. B Cells contribute to neuroinflammation by producing autoantibodies, presenting antigens, and secreting pro-inflammatory cytokines (Mayne et al., 2020; Müller et al., 2025; Sanmarco et al., 2021; Vukovic et al., 2024; Xie et al., 2015).

Thus, the interplay between CNS-resident and infiltrating immune cells determines the trajectory of neuroinflammation. While certain responses are essential for defense and repair, prolonged or maladaptive activation exacerbates neuronal damage and accelerates neurodegeneration. Understanding these cellular interactions provides valuable insights into potential therapeutic strategies aimed at restoring immune balance and mitigating neuroinflammatory disease progression.

## 3 Molecular mediators of neuroinflammation

Neuroinflammation is driven by a complex network of molecular mediators (Figure 1) that coordinate communication between CNS-resident cells and infiltrating immune cells (Alboni and Maggi, 2015; Amanollahi et al., 2023; Costagliola et al., 2022; Di Benedetto et al., 2017; Garg et al., 2009; Goshen et al., 2008; Guarnieri et al., 2020; Ifergan and Miller, 2020; Kveler et al., 2018; Müller et al., 2025). These mediators, including cytokines, chemokines, extracellular vesicles, and lipid metabolites, regulate inflammatory responses, either promoting resolution and repair (Figure 1, left) or sustaining chronic inflammation and neuronal damage (Figure 1, right). Understanding their roles is crucial for developing targeted therapies to modulate neuroinflammation in chronic neurodegenerative diseases.

Cytokines are key regulators of immune signaling in the CNS, mediating both pro-inflammatory (Figure 1, right) and anti-inflammatory processes (Figure 1, left). The balance between these opposing signals determines whether neuroinflammation resolves or persists, contributing to neurodegeneration (Garg et al., 2009; Guarnieri et al., 2020; Kveler et al., 2018; Müller et al., 2025). Pro-inflammatory cytokines, such as TNF-*α*, IL-1*β*, and IL-6, are primarily secreted by activated microglia and astrocytes in response to injury or pathogenic stimuli. TNF-α enhances microglial activation, induces oxidative stress, and promotes synaptic dysfunction. IL-1β, a potent inducer of inflammation, stimulates the release of additional cytokines and disrupts neuronal homeostasis.

On the other hand, cytokines can have dual roles, with their effects depending on timing, intensity, and cellular context. For example, TNF-α, traditionally viewed as a pro-inflammatory cytokine, also plays complex roles in modulating synaptic functions. This mechanism is crucial for maintaining neural circuit stability. Conversely, elevated levels of TNF-α disrupts long-term potentiation (LTP) in the hippocampus, impairing learning, memory, and cognitive functions. Similarly, IL-6 plays a dual role, contributing to neuroinflammation in acute settings but also supporting neuronal survival under certain conditions (Alboni and Maggi, 2015; Gonzalez Caldito, 2023; Heir and Stellwagen, 2020; Prieto and Cotman, 2017; Schneider et al., 1998).

Anti-inflammatory cytokines, such as IL-10 and TGF-*β*, act usually as counter-regulatory signals that suppress excessive inflammation and promote tissue repair (Li and Flavell, 2008). IL-10 inhibits pro-inflammatory cytokine production and enhances the clearance of cellular debris by microglia. TGF-β regulates glial activation, supports neuronal survival, and modulates immune cell infiltration into the CNS. However, in chronic neuroinflammatory diseases, the protective effects of these cytokines may be insufficient to counteract persistent inflammatory signaling (Lobo-Silva et al., 2016; Norden et al., 2014).

Chemokines are small signaling proteins that navigate immune cell migration, recruitment, and positioning within the CNS. They create chemotactic gradients that direct the movement of microglia, astrocytes, and infiltrating peripheral immune cells in response to inflammatory cues (Reaux-Le Goazigo et al., 2013; Williams et al., 2014). Pro-inflammatory chemokines, such as CCL2 (MCP-1), CCL5 (RANTES), and CXCL10 (IP-10), facilitate the recruitment of monocytes, macrophages, and T cells into the CNS during neuroinflammatory conditions (Roberts et al., 2015; Sørensen et al., 2002). CCL2 is upregulated in neurodegenerative diseases, promoting the infiltration of peripheral immune cells that exacerbate inflammation. CXCL10, secreted by astrocytes and microglia, attracts activated T cells and sustains the inflammatory environment (Clarner et al., 2015; Koper et al., 2018).

Homeostatic and anti-inflammatory chemokines, such as CX3CL1 (fractalkine), maintain CNS immune balance under physiological conditions. Fractalkine signaling via its receptor, CX3CR1, modulates microglial activity, promoting their neuroprotective functions and limiting excessive inflammation. Disruptions in this signaling axis have been implicated in the progression of neurodegenerative diseases (Pawelec et al., 2020; Subbarayan et al., 2022).

Extracellular vesicles (EVs), including exosomes and microvesicles, play a crucial role in intercellular communication by transferring proteins, lipids, and RNA molecules between cells (Buzas, 2023). These vesicles serve as messengers in neuroinflammation, influencing microglial and astrocytic responses, as well as neuronal survival (De Masi et al., 2024). In neuroinflammatory diseases, microglia and astrocytes release EVs enriched in inflammatory cytokines, miRNAs, and toxic protein aggregates, such as amyloid-β and alpha-synuclein. These EVs propagate inflammatory signals and contribute to disease pathology by promoting synaptic dysfunction, neuronal apoptosis, and glial reactivity. Conversely, some EVs carry anti-inflammatory and neuroprotective cargo, facilitating tissue repair and immune resolution. The dual nature of EV-mediated signaling makes them promising targets for therapeutic intervention, either by inhibiting harmful EV release or engineering vesicles for targeted drug delivery (Du et al., 2023; Picca et al., 2022).

Lipid mediators are lipid-derived signaling molecules, such as prostaglandins, leukotrienes, and specialized pro-resolving mediators (SPMs), that play a crucial role in regulating and modulating neuroinflammatory responses (Basil and Levy, 2016). Pro-inflammatory lipid mediators, such as prostaglandin E2 (PGE2) and leukotriene B4 (LTB4), are produced by activated microglia and astrocytes in response to inflammatory stimuli. PGE2 amplifies neuroinflammatory signaling by enhancing cytokine production and disrupting synaptic function. LTB4 promotes immune cell recruitment and sustains chronic inflammation in neurodegenerative diseases. SPMs, including lipoxins, resolvins, and maresins, counteract inflammation and support tissue repair (Schebb et al., 2022; Serhan, 2014). These lipid mediators suppress microglial activation, enhance phagocytic clearance of toxic debris, and promote neuronal survival (Schebb et al., 2022). Impaired SPM production has been observed in neurodegenerative diseases, suggesting that enhancing their availability could be a potential therapeutic strategy.

Additionally, oxidative stress plays a critical role in neuroinflammation, with reactive oxygen species (ROS) and reactive nitrogen species (RNS) contributing to cellular damage. Microglia and astrocytes produce ROS in response to inflammatory stimuli, leading to mitochondrial dysfunction, lipid peroxidation, and neuronal death (Kalyanaraman et al., 2024; Kempuraj et al., 2016). Chronic oxidative stress exacerbates neuroinflammatory signaling, creating a vicious cycle that accelerates disease progression (Teleanu et al., 2022).

Thus, in chronic neuroinflammatory conditions, the balance between pro-and anti-inflammatory mediators becomes disrupted, supporting a harmful environment that promotes neurodegeneration. In AD, elevated levels of TNF-*α*, IL-1β, and chemokines contribute to persistent microglial activation and synaptic loss. In PD, inflammatory cytokines enhance alpha-synuclein aggregation and dopaminergic neuron degeneration. In MS, chemokine-driven immune cell infiltration leads to demyelination and axonal injury (Chamera et al., 2020; Leng and Edison, 2021; Wendimu and Hooks, 2022; Yildirim-Balatan et al., 2024). In the following section, we will focus on the critical role of microglia in driving and regulating neuroinflammation, exploring their diverse functions and impact on neurodegenerative processes.

## 4 Role of microglia in chronic neuroinflammation

Microglia exist in a dynamic continuum of functional states rather than a rigid pro-inflammatory (M1) vs. anti-inflammatory (M2) binary. In homeostatic conditions, microglia actively surveil their environment, support synaptic remodeling, and clear cellular debris. Upon encountering inflammatory stimuli, they shift into reactive states, which can be protective or detrimental depending on the context (Haidar et al., 2022; Müller et al., 2025; Nosi et al., 2021; Woodburn et al., 2021). While classically activated microglia (affiliated to M1) release pro-inflammatory cytokines (e.g., TNF-α, IL-1β) and ROS, alternatively activated microglia (affiliated to M2) promote tissue repair and clearance of toxic aggregates. However, these categories oversimplify microglial heterogeneity. Single-cell RNA sequencing has revealed disease-associated microglial (DAM), lipid-droplet-accumulating microglial (LDAM), and neurodegenerative microglial (MGnD) phenotypes, which display distinct transcriptomic profiles depending on the neuroinflammatory milieu (Nosi et al., 2021; Paolicelli et al., 2022; Ransohoff, 2016; Wendimu and Hooks, 2022).

Emerging microglial subpopulations arise in response to specific pathological stimuli, providing valuable insights into microglial heterogeneity. One such subpopulation, DAM, has been identified in neurodegenerative conditions like AD. DAM exhibit a distinct transcriptional profile regulated by triggering receptor expressed on myeloid cells 2 (TREM2) and apolipoprotein E (APOE)-dependent pathways. This activation allows them to engage with amyloid-beta plaques, damaged neurons, and other neurodegenerative hallmarks through processes such as phagocytosis and the release of inflammatory mediators. Depending on the context, DAM can play both protective and potentially harmful roles (Chen et al., 2024; Dias and Socodato, 2025; Gratuze et al., 2023; Haney et al., 2024; Hou et al., 2022; Paolicelli et al., 2022).

Similarly, LDAM are associated with aging and metabolic disturbances. Characterized by intracellular lipid droplet accumulation, LDAM exhibit impaired phagocytosis and an altered inflammatory response. These microglia produce elevated levels of pro-inflammatory cytokines and oxidative stress markers, contributing to chronic neuroinflammation in aging brains. Their presence has also been linked to neurodegenerative diseases and metabolic disorders, highlighting their broader relevance in both systemic and central nervous system pathologies (Haney et al., 2024; Paolicelli et al., 2022; Wei and Li, 2022).

MGnD represent a distinct microglial state associated with neurodegenerative diseases, particularly in response to neuronal damage and metabolic stress. These cells undergo a transcriptional shift marked by the downregulation of homeostatic genes and the upregulation of pathways involved in lipid metabolism, phagocytosis, and inflammation. MGnD are thought to play a dual role, contributing to debris clearance and tissue repair while also amplifying inflammatory responses that may exacerbate disease progression. Their emergence is influenced by TREM2 signaling, positioning them as key players in neurodegenerative pathologies such as AD (Gao et al., 2023; Wei and Li, 2022). These findings highlight the adaptability of microglia, enabling them to assume distinct functional states in response to environmental signals such as cytokine patterns, tissue injury, and metabolic stress.

Remarkably, chronic neuroinflammation can lead to microglial priming, a state in which microglia become hypersensitive to subsequent stimuli. Primed microglia exhibit exaggerated inflammatory responses upon secondary activation, contributing to the persistence of neuroinflammation in chronic diseases (Perry and Holmes, 2014). This phenomenon has been implicated in aging, where low-grade inflammation primes microglia, leading to heightened reactivity in neurodegenerative conditions. Additionally, microglial memory, shaped by previous inflammatory encounters, influences their response patterns, potentially exacerbating or dampening disease progression depending on prior exposures (Norden et al., 2015; Perry and Holmes, 2014).

The question of whether neuroinflammation is predominantly driven by peripheral immune signals or by CNS-resident cells remains a critical debate in the field. Some studies suggest that systemic inflammation (e.g., infections, metabolic disorders) can drive neuroinflammation by BBB integrity. Once the BBB is disrupted, peripheral immune cells, cytokines, and other inflammatory mediators can infiltrate the CNS, triggering microglial activation. This mechanism has been implicated in neurodegenerative diseases like AD, PD, and MS (Basilico et al., 2022; Muzio et al., 2021; Tahmasebi and Barati, 2023). Others argue that microglia intrinsically regulate neuroinflammation in response to CNS damage independent of peripheral influences. CNS-resident immune cells, particularly microglia and astrocytes, are capable of sensing and amplifying local inflammatory signals, driving pathology even in the absence of systemic inflammation (Dias and Socodato, 2025). For example, in AD, microglia are activated by amyloid-*β* accumulation, even before BBB breakdown occurs (Dias and Socodato, 2025; Feng et al., 2020).

Many studies have investigated the effects of removing microglia in various neuroinflammatory conditions caused by peripheral or central insults. Approaches include pharmacological depletion using CSF1R inhibitors such as PLX5622, genetic ablation models, and selective inhibition of microglial activation (Basilico et al., 2022; Coleman et al., 2020; Dagher et al., 2015; Tahmasebi and Barati, 2023; Vichaya et al., 2020). The findings underscore the complexity of neuroimmune interactions, where microglia function at the intersection of peripheral and central inflammatory processes.

### 4.1 Microglia–neuron crosstalk

Microglia and neurons engage in constant bidirectional communication, which is crucial for synaptic maintenance and neuronal survival (Müller et al., 2025; Soteros and Sia, 2022). This interaction becomes dysregulated in chronic neuroinflammatory diseases, leading to impaired synaptic function and neurodegeneration. During development and in homeostatic conditions, microglia mediate synaptic pruning by engulfing excess or weak synapses, a process essential for neural circuit refinement (Hu and Tao, 2024). However, in disease states, excessive microglial-mediated pruning leads to synaptic loss, contributing to cognitive decline in disorders such as AD. Disruptions in complement signaling (e.g., C1q, C3) enhance aberrant synapse elimination, particularly in early disease stages (Batista et al., 2024; Hu and Tao, 2024; Norden et al., 2015).

On the other hand, neurons regulate microglial activity through signaling pathways such as CX3CL1-CX3CR1 (fractalkine signaling) and CD200-CD200R. In neurodegenerative diseases, downregulation of these signals leads to uncontrolled microglial activation, promoting neurotoxic cascades. Activated microglia release inflammatory cytokines, ROS, and nitric oxide (NO), exacerbating neuronal stress and synaptic dysfunction (Chamera et al., 2020; Gao et al., 2023). In PD for instance, microglial-derived TNF-*α* and IL-1β enhance alpha-synuclein aggregation, further activating microglia and perpetuating a cycle of neurodegeneration (Yildirim-Balatan et al., 2024).

### 4.2 Microglia-astrocyte interactions

Microglia and astrocytes function as key regulators of neuroinflammation, engaging in reciprocal interactions that amplify or modulate inflammatory responses. Activated microglia release cytokines such as IL-1β and TNF-α, which induce astrocytic reactivity. Reactive astrocytes, in turn, release additional pro-inflammatory mediators, including complement proteins (e.g., C3) and chemokines, sustaining chronic inflammation. This feedback loop is evident in AD, where microglia-induced astrocytic activation contributes to widespread neuroinflammation and synaptic dysfunction (DiSabato et al., 2016; Kwon and Koh, 2020; Lian et al., 2016).

Astrocytes exhibit a spectrum of reactive phenotypes, broadly categorized as neurotoxic (A1) or neuroprotective (A2). Microglia influence this polarization through cytokine signaling. For instance, microglia-derived IL-1α, TNF-α, and C1q drive astrocytes toward an A1 phenotype, which exacerbates neuronal damage. Conversely, microglia can promote neuroprotective astrocytes by secreting anti-inflammatory factors such as TGF-β (DiSabato et al., 2016; Müller et al., 2025; Sanmarco et al., 2021). Targeting microglia-astrocyte crosstalk may provide therapeutic opportunities to modulate neuroinflammation in neurodegenerative diseases.

### 4.3 Implications for disease pathology

Microglial dysfunction plays a central role in the pathogenesis of multiple neurodegenerative diseases. Disease-specific alterations in microglial activity contribute to chronic neuroinflammation and neuronal loss. In neurodegenerative diseases, disruption of the BBB allows peripheral immune cells, such as monocytes, macrophages, and T cells, to infiltrate the CNS. Microglia play a key role in shaping these infiltrating immune responses (Di Benedetto et al., 2017; Müller et al., 2025; Woodburn et al., 2021). In MS for example, microglia facilitate the recruitment of autoreactive T cells that attack myelin, exacerbating demyelination and axonal loss (Dong and Yong, 2019). In AD and PD, microglia contribute to BBB dysfunction by releasing inflammatory cytokines that increase vascular permeability, allowing additional immune cells to enter the CNS and sustain inflammation (Murdaca et al., 2022; Takata et al., 2021).

In AD, microglia fail to effectively clear amyloid-beta (Aβ) plaques, leading to chronic inflammation and neurotoxicity (Leng and Edison, 2021; Wendimu and Hooks, 2022). Disease-associated microglia, known as DAM (Deczkowska et al., 2018), upregulate phagocytic pathways but remain inefficient at plaque clearance, while excessive complement-mediated synapse elimination contributes to cognitive decline (Leng and Edison, 2021; Rachmian et al., 2024). In PD, microglia induce neuroinflammation through the release of TNF-*α* and IL-1β, which promote dopaminergic neuron degeneration in the substantia nigra. Alpha-synuclein aggregates trigger microglial activation, perpetuating a cycle of neurotoxicity (Wendimu and Hooks, 2022; Yildirim-Balatan et al., 2024).

In MS, microglia contribute to demyelination and axonal injury by producing pro-inflammatory cytokines and oxidative stress mediators. Additionally, they modulate T-cell responses, influencing the severity of autoimmune neuroinflammation. Upon injury, oligodendrocytes release myelin debris, which can be phagocytosed by microglia. However, excessive debris accumulation in MS leads to persistent microglial activation and impaired remyelination (Dong and Yong, 2019; Gao et al., 2023; Lopez-Muguruza and Matute, 2023; Saez-Calveras and Stuve, 2022; Yeung et al., 2019).

Thus, microglia serve as key regulators of neuroinflammation, with functions spanning from neuroprotection to neurotoxicity, depending on the context and stage of inflammation. Their interactions with neurons and astrocytes critically shape disease progression in neurodegenerative conditions.

## 5 Intercellular communication in neuroinflammation

Neuroinflammation is orchestrated by complex intercellular communication networks involving both resident CNS cells and infiltrating peripheral immune cells (Müller et al., 2025; Nosi et al., 2021). These interactions occur through diverse mechanisms, including paracrine signaling, direct cell–cell contact, and systemic immune modulation. In chronic neuroinflammatory diseases, dysregulated intercellular communication perpetuates inflammatory states, exacerbating neuronal dysfunction and disease progression. This section explores the key mechanisms of local and peripheral immune-CNS communication and how their dysregulation contributes to chronic inflammation.

### 5.1 Local CNS communication

Within the CNS, resident cells—including microglia, astrocytes, oligodendrocytes, and neurons—communicate through paracrine signaling and direct intercellular interactions. These mechanisms regulate immune responses, neuronal function, and tissue homeostasis but become disrupted in chronic neuroinflammation (Kveler et al., 2018; Müller et al., 2025).

Paracrine signaling enables CNS-resident cells to communicate through secreted molecules such as cytokines, chemokines, growth factors, and extracellular vesicles. This mechanism is essential for maintaining homeostasis and regulating responses to injury and neurodegeneration (Decrock et al., 2015). Microglia play a central role by releasing pro-and anti-inflammatory cytokines (e.g., TNF-*α*, IL-1β, IL-10), influencing astrocytes, neurons, and other microglia. Astrocytes, in turn, release gliotransmitters and cytokines that modulate neuronal activity and inflammatory cascades. Oligodendrocyte precursor cells rely on paracrine signals for differentiation and myelination. Neurons contribute by releasing neurotrophic factors (e.g., BDNF, NGF) that support glial function but can also trigger inflammatory responses through excessive glutamate release (Kveler et al., 2018; Lian et al., 2016; Santello et al., 2019; Song and Dityatev, 2018). The balance of these signals determines whether neuroinflammation remains controlled or becomes chronic and harmful (Vaz et al., 2022).

Another mechanism of intercellular communication in the CNS involves direct physical interactions, including gap junctions and receptor-ligand binding (Orellana et al., 2013; Sarapultsev et al., 2023). Gap junctions, composed of connexin proteins, form channels that allow the exchange of ions, metabolites, and signaling molecules between adjacent cells, ensuring rapid coordination of cellular responses (Decrock et al., 2015; Lovinger, 2008; Orellana et al., 2013). Astrocytes establish extensive gap junction networks, supporting metabolic coupling and ion homeostasis. Microglia and astrocytes communicate through both gap junctions and surface receptor interactions, modulating immune responses. Neurons also utilize gap junctions for electrical coupling, particularly in early development and injury responses. While crucial for CNS function, dysregulated gap junction signaling can exacerbate neuroinflammation and contribute to neurodegeneration (Kielian, 2008; Orellana et al., 2013). For instance, dysregulated gap junction signaling can amplify neuroinflammation by enabling the uncontrolled spread of pro-inflammatory signals, disrupting ion homeostasis, and enhancing glial activation, which may contribute to chronic neurodegenerative processes (Kielian, 2008). Modulating these direct communication pathways may therefore present potential therapeutic strategies for controlling inflammation and protecting neuronal health.

### 5.2 Communication pathways between peripheral immunity and the brain

While the brain has historically been considered immune-privileged, emerging evidence highlights bidirectional communication between the CNS and the peripheral immune system (Di Benedetto et al., 2017; Müller and Di Benedetto, 2024; Nosi et al., 2021; Takata et al., 2021). Disruptions in these interactions contribute to chronic neuroinflammation and neurodegenerative disease progression. Under normal conditions, the BBB tightly regulates the entry of immune cells into the brain (Figure 1, left). However, in chronic neuroinflammation, BBB integrity is compromised, allowing infiltration of peripheral immune cells (Figure 1, right). Inflammatory cytokines such as TNF-*α* and IL-6 disrupt tight junction proteins (e.g., occludin, claudins), increasing BBB permeability (Kempuraj et al., 2016; Sanmarco et al., 2021; Takata et al., 2021). In MS, this facilitates the entry of autoreactive T cells that attack myelin. Chemokines such as CCL2 and CXCL12 recruit monocytes and lymphocytes across the BBB. In PD, infiltrating monocytes contribute to dopaminergic neuron loss in the substantia nigra (Dong and Yong, 2019; Koper et al., 2018; Lopez-Muguruza and Matute, 2023; Sørensen et al., 2002; Yeung et al., 2019).

Recent discoveries reveal that the CNS communicates with peripheral immunity via meningeal lymphatic vessels, which facilitate antigen presentation and immune surveillance (Guo et al., 2023; Li et al., 2022). Impaired drainage of inflammatory mediators through these vessels exacerbates neuroinflammatory diseases. Furthermore, peripheral inflammation can worsen CNS pathology through both humoral and cellular mechanisms, some of which are briefly outlined below:

- Cytokine-induced neuroinflammation: systemic infections and chronic inflammatory conditions (e.g., diabetes, obesity) elevate circulating cytokines such as IL-1β and TNF-α, which cross the BBB and activate microglia. Epidemiological studies link chronic systemic inflammation to increased risk of neurodegenerative diseases (Moyse et al., 2022; Murdaca et al., 2022; Takata et al., 2021; Wyss-Coray and Mucke, 2002).
- Gut-brain axis and microbiota: dysbiosis of the gut microbiome influences CNS inflammation through microbial metabolites and *vagus nerve* signaling. In multiple sclerosis, altered gut microbiota composition is associated with disease progression and immune dysregulation (Silva et al., 2020; Wrona et al., 2024).
- Aging-related immune changes: age-related alterations in peripheral immune cells, such as senescent T cells and myeloid skewing, enhance neuroinflammation and contribute to cognitive decline (Di Benedetto et al., 2017; Müller and Di Benedetto, 2023; Müller and Di Benedetto, 2024; Wrona et al., 2024).

In summary, intercellular communication plays a crucial role in neuroinflammation, integrating signals from CNS-resident cells and peripheral immune responses. In chronic neuroinflammatory diseases, dysregulated signaling networks sustain inflammation and exacerbate neuronal damage. Targeting these maladaptive communication pathways offers a promising avenue for therapeutic interventions aimed at restoring CNS immune homeostasis and mitigating neurodegenerative disease progression.

## 6 Modulating neuroinflammation: therapeutic perspectives

**Figure 2:**
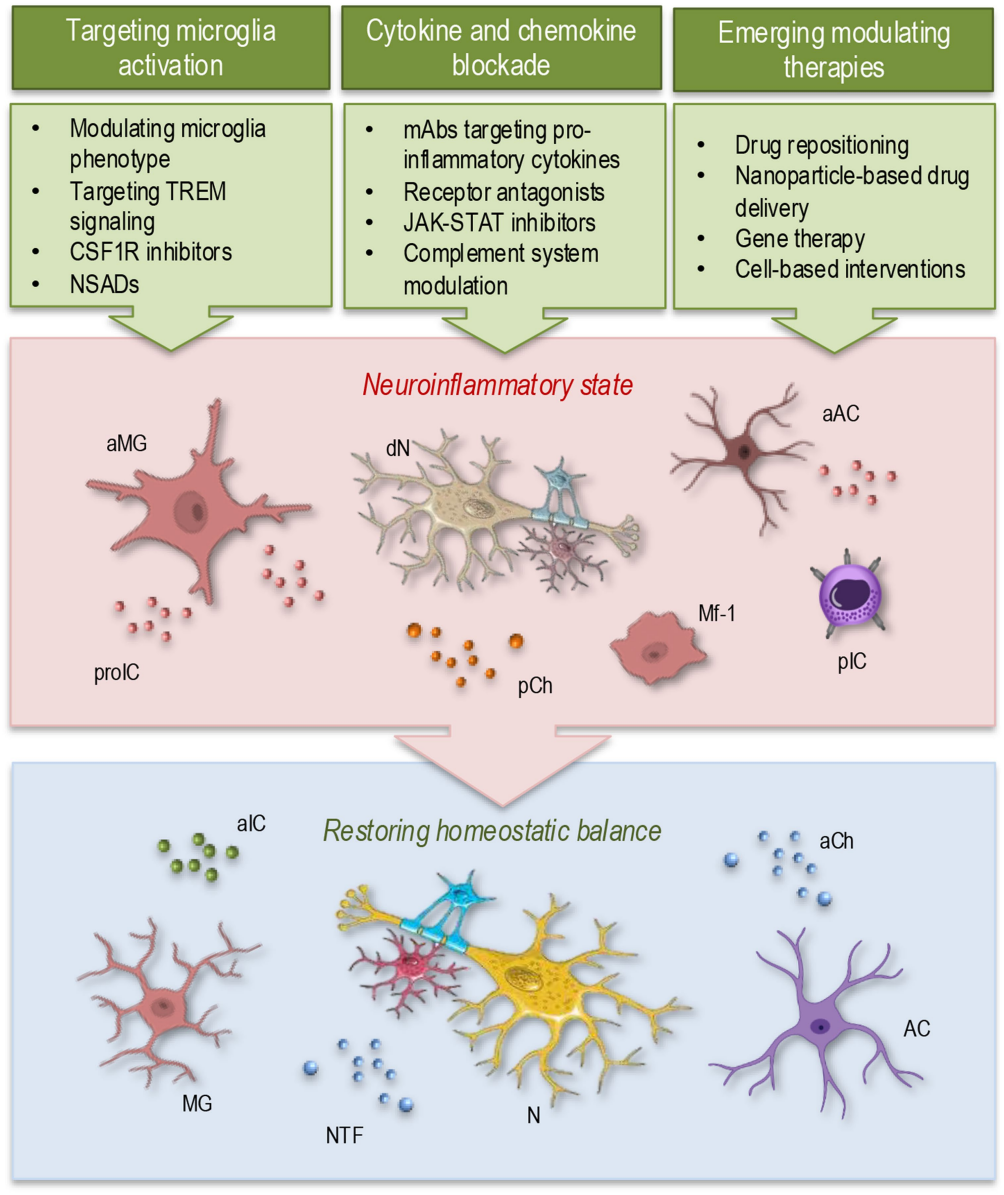
Therapeutic modulation of neuroinflammation. Therapeutic strategies aimed at regulating microglial activity and immune responses hold potential for restoring homeostatic balance in the CNS. Approaches such as microglial reprogramming by targeting microglia activation, inhibition of pro-inflammatory cytokines and chemokines, and advanced treatments—including nanoparticle-based drug delivery, gene therapy, and cell-based interventions—may offer innovative avenues for modulating neuroinflammation and restoring homeostasis. aMG, activated microglia; dN, degenerating neuron; aAC, activated astrocyte; proIC, pro-inflammatory cytokines; pCh, pro-inflammatory chemokines; Mf-1, macrophage type 1; pIC, peripheral immune cell; aIC, anti-inflammatory cytokines; aCh, anti-inflammatory chemokines; AC, astrocyte; MG, microglia; NTF, neurotrophic factors; N, neuron.

Given the central role of neuroinflammation in the progression of chronic neurodegenerative diseases, therapeutic strategies aimed at modulating immune responses within the CNS are of increasing interest. Effective interventions must restore immune homeostasis, mitigate neurotoxic inflammation, and preserve essential defense mechanisms (Figure 2). This section provides a brief overview of current and emerging approaches to targeting neuroinflammation, along with the challenges and future directions in developing successful therapies.

### 6.1 Targeting microglial activation

As discussed earlier, microglia play a central role in regulating neuroinflammation, with their activation state profoundly impacting disease pathology. Therapeutic strategies (Figure 2, green) focus on shifting microglia from a chronic pro-inflammatory phenotype (Figure 2, red) toward a neuroprotective state (Figure 2, blue). Listed below are some examples illustrating these strategies:

- Modulating microglial phenotypes: small molecules such as minocycline, rapamycin, and PPAR-*γ* agonists (e.g., pioglitazone) have been explored for their ability to suppress excessive microglial activation and promote repair-associated phenotypes (Lin et al., 2023; Pinto et al., 2016; Wang et al., 2023; Xue et al., 2024).
- Targeting TREM2 signaling: TREM2 is crucial for microglial phagocytosis and homeostasis. Agonistic antibodies and TREM2-boosting compounds are being investigated to enhance microglial resilience in diseases such as Alzheimer’s and Parkinson’s (Dhapola et al., 2021; Xue et al., 2024).
- CSF1R inhibitors: colony-stimulating factor 1 receptor (CSF1R) signaling regulates microglial survival and proliferation. Selective CSF1R inhibitors have shown promise in limiting excessive microgliosis in neurodegenerative conditions (Johnson et al., 2023; Spangenberg et al., 2019).
- Re-purposing anti-inflammatory drugs: nonsteroidal anti-inflammatory drugs (NSAIDs) and corticosteroids have been evaluated for their effects on microglial function, though their clinical efficacy remains variable (Ajmone-Cat et al., 2010; Dhapola et al., 2021).

### 6.2 Cytokine and chemokine blockade

Excessive production of pro-inflammatory cytokines and chemokines perpetuates chronic neuroinflammation. Therapeutic strategies aim to neutralize these mediators to limit neurotoxic immune responses. The following are just a few examples that offer insight into these strategies:

- Monoclonal antibodies (mAbs): antibodies targeting pro-inflammatory cytokines, such as anti-TNF-α (infliximab, etanercept) and anti-IL-1β (canakinumab), have been explored in neurological conditions (Costagliola et al., 2022; Dhapola et al., 2021).
- Receptor antagonists: IL-1 receptor antagonist (anakinra) and CCR2/CCR5 antagonists have shown potential in reducing leukocyte infiltration and microglial activation in preclinical models (Aronica et al., 2017; Ifergan and Miller, 2020).
- JAK–STAT pathway inhibitors: Janus kinase (JAK) inhibitors, such as tofacitinib, suppress inflammatory cytokine signaling and may be beneficial in neuroinflammatory disorders (Jain et al., 2021; Shariq et al., 2018).
- Complement system modulation: targeting complement proteins (e.g., C3 and C5 inhibitors) is a promising approach to limit excessive immune activation, particularly in conditions like multiple sclerosis (Saez-Calveras and Stuve, 2022; Zelek and Morgan, 2022).

### 6.3 Emerging therapies for modulating neuroinflammation

New therapeutic strategies are being developed to improve the precision and effectiveness of neuroinflammation treatment. For example, the drug repositioning is a promising strategy for drug discovery, involving the identification of new therapeutic applications for existing drugs that have already been approved for other clinical uses and have established safety and tolerability (Pillaiyar et al., 2020). Given these advantages, drug repurposing serves as a valuable approach in the search for new treatments for neurodegenerative diseases (Munafo et al., 2020).

Another promising approach involves nanoparticle-based drug delivery, which enhances blood–brain barrier penetration while minimizing systemic side effects (Huang et al., 2024). Lipid-based nanoparticles can transport anti-inflammatory agents, such as dexamethasone or siRNA, directly to activated microglia. Polymeric and magnetic nanoparticles offer additional advantages, with polymeric carriers encapsulating cytokine inhibitors and magnetic nanoparticles enabling targeted drug release through external fields (Du et al., 2023; Huang et al., 2024; Zhu et al., 2021).

Additional emerging strategy is gene therapy, which leverages advanced gene-editing tools to regulate inflammatory pathways. CRISPR technology is being explored to modify pro-inflammatory genes in glial cells, while adeno-associated virus (AAV) vectors serve as carriers for anti-inflammatory genes or RNA molecules that help restore immune balance (Datta et al., 2023; Lin et al., 2025).

Cell-based therapies also show potential in modulating neuroinflammation. Stem cell therapy, using mesenchymal or neural stem cells, has demonstrated immunomodulatory effects in conditions like MS and PD (Isakovic et al., 2023). Additionally, engineered immune cells, such as CAR-T cells—originally designed for cancer treatment—are being investigated for their ability to target dysfunctional immune responses in neurodegenerative diseases (Vukovic et al., 2024).

These innovative approaches represent promising avenues for controlling neuroinflammation and mitigating its harmful effects in neurodegenerative disorders.

### 6.4 Challenges in the field and future directions

Despite significant advances in understanding and targeting neuroinflammation, several challenges remain. One key issue is balancing inflammation control with immune function preservation. While suppressing neuroinflammation is crucial for preventing neuronal damage, excessive immune suppression can compromise the CNS’s ability to repair tissue and defend against infections.

Another challenge lies in the heterogeneity of neuroinflammatory responses. Inflammatory mechanisms vary across different neurodegenerative diseases and patient populations, making it difficult to develop one-size-fits-all therapies. The neuroinflammation plays dual role in the CNS, acting as both a protective and pathological mechanism depending on the context. The interplay between chronic triggers and impaired resolution mechanisms highlights the need for therapeutic strategies that fine-tune, rather than completely suppress, neuroinflammation. Future research should focus on identifying biomarkers that distinguish beneficial from detrimental immune responses to guide more targeted interventions.

Additionally, translating preclinical findings into clinical success remains a major hurdle. Many treatments that show promise in animal models fail in human trials due to differences in neuroimmune interactions and blood–brain barrier permeability. The extrapolating findings from animal models to human conditions remains a significant challenge in neuroinflammation research. While rodent models have provided crucial mechanistic insights, fundamental differences in immune system composition, cytokine signaling, and brain architecture can lead to discrepancies in disease progression and treatment responses. These limitations contribute to the frequent failure of preclinical therapies in clinical trials.

To address this translational gap, integrating complementary approaches such as human iPSC-derived microglia, organoid models, and *in vivo* imaging is essential. Additionally, leveraging patient-derived data from postmortem brain tissue, cerebrospinal fluid, and blood-based biomarkers can provide a more accurate understanding of neuroimmune interactions in humans. Combining these methods with advanced computational modeling may help refine predictions and improve the success of translational research.

Variability in experimental approaches presents a major challenge in neuroinflammation research. Differences in disease models, neuroinflammation induction methods, and timing of sample collection can lead to conflicting findings. For example, the choice of genetic models, toxin exposure, or systemic inflammation paradigms influences immune responses, making direct comparisons difficult. Analytical methods further contribute to discrepancies. Bulk RNA sequencing masks cellular heterogeneity, while single-cell approaches offer higher resolution but introduce processing artifacts. Similarly, imaging and flow cytometry differ in sensitivity and spatial insights.

These methodological differences complicate the interpretation of microglial activation states and inflammatory marker expression. To improve reproducibility, standardization of protocols, transparent reporting, and multimodal validation of findings are essential. Cross-species comparisons, meta-analyses, and integrative approaches can help reconcile inconsistencies and advance a more unified understanding of neuroimmune dynamics.

The interaction between peripheral immune signals and CNS-driven mechanisms in regulating microglial activation remains a topic of debate, highlighting the complex interplay between systemic inflammation and local neuroimmune responses. This debate directly affects experimental models and treatment strategies. If peripheral immune activation is the primary trigger, therapies targeting systemic inflammation, the gut microbiome, or BBB integrity might be prioritized. Conversely, if CNS-resident microglia are the main drivers, interventions focusing on modulating microglial states, preventing chronic activation, or enhancing anti-inflammatory functions become more relevant. Therefore, understanding the specific conditions under which peripheral signals dominate microglial activation versus when intrinsic CNS mechanisms take precedence is critical for developing effective therapeutic strategies.

While innovative therapeutic strategies such as nanoparticle-based drug delivery, gene therapy, and cell-based interventions hold promise for mitigating neuroinflammation, their translational success remains uncertain. Many of these approaches have demonstrated efficacy in preclinical models, yet the complexity of human neuroimmunology and long-term safety concerns pose significant challenges. Optimism surrounding these emerging therapies must be tempered with rigorous clinical validation, ensuring that efficacy observed in animal models translates into meaningful therapeutic benefits in patients.

Future research should prioritize controlled clinical trials, standardized methodologies, and long-term follow-ups to assess both efficacy and potential adverse effects, ultimately refining therapeutic strategies for neurodegenerative diseases. Precision medicine, guided by patient-specific inflammatory profiles, could help tailor interventions, while combining multiple therapeutic modalities may improve treatment outcomes. Addressing these challenges will be essential for developing more effective neuroinflammation-targeting therapies in neurodegenerative diseases.

## 7 Conclusion

Chronic neuroinflammation is a hallmark of many neurodegenerative diseases, driven by complex intercellular communication networks involving resident CNS cells and infiltrating immune cells. The interplay between microglia, astrocytes, neurons, and peripheral immune components shapes the inflammatory landscape, influencing disease progression and neuronal survival. Aberrant signaling loops, sustained cytokine production, and disrupted neuroimmune interactions perpetuate inflammation, contributing to neurodegeneration in conditions such as AD, PD, and MS.

Targeting microglial activity and modulating immune responses present promising therapeutic opportunities to restore homeostatic balance in the CNS. Strategies such as reprogramming microglia, blocking pro-inflammatory cytokines, and leveraging emerging therapies—including nanoparticle-based drug delivery, gene therapy, and cell-based interventions—offer innovative approaches to mitigating neuroinflammation. However, challenges remain in achieving therapeutic efficacy while preserving essential immune functions, translating preclinical findings into clinical applications, and addressing the heterogeneity of neuroinflammatory responses.

To fully unravel the complexity of neuroinflammatory processes, integrative research approaches are essential. Advancements in single-cell transcriptomics, spatial imaging, and systems biology will enhance our understanding of cell-type-specific responses and intercellular signaling in chronic neuroinflammation. Collaborative efforts across neuroscience, immunology, and biotechnology will be crucial in developing precise, personalized therapeutic interventions. By bridging mechanistic insights with translational applications, future research holds the potential to transform the treatment of neurodegenerative diseases and improve patient outcomes.
